# Clinical Impact of Sarcopenia Screening on Long‐Term Mortality in Patients Undergoing Coronary Bypass Grafting

**DOI:** 10.1002/jcsm.13645

**Published:** 2024-11-08

**Authors:** Seung Hun Lee, Jinhwan Jo, Jeong Hoon Yang, Sung Mok Kim, Ki Hong Choi, Young Bin Song, Dong Seop Jeong, Joo Myung Lee, Taek Kyu Park, Joo‐Yong Hahn, Seung‐Hyuk Choi, Su Ryeun Chung, Yang Hyun Cho, Kiick Sung, Wook Sung Kim, Hyeon‐Cheol Gwon, Young Tak Lee

**Affiliations:** ^1^ Department of Internal Medicine, Division of Cardiology, Heart Center, Chonnam National University Hospital Chonnam National University Medical School Gwangju South Korea; ^2^ Department of Internal Medicine, Division of Cardiology, Heart Vascular Stroke Institute, Samsung Medical Center Sungkyunkwan University School of Medicine Seoul South Korea; ^3^ Department of Radiology, Cardiovascular Imaging Center, Heart Vascular Stroke Institute, Samsung Medical Center Sungkyunkwan University School of Medicine Seoul South Korea; ^4^ Department of Thoracic and Cardiovascular Surgery, Heart Vascular Stroke Institute, Samsung Medical Center Sungkyunkwan University School of Medicine Seoul South Korea

**Keywords:** coronary artery bypass grafting, coronary artery disease, frailty, prognosis, sarcopenia

## Abstract

**Background:**

Sarcopenia is an aging‐related condition characterized by loss of skeletal muscle mass and is an indicator of subclinical atherosclerosis. The relationship between reduced muscle mass and long‐term clinical outcomes in patients with advanced coronary artery disease who have undergone coronary artery bypass grafting (CABG) is not fully understood. This study is sought to evaluate the prognostic implications of sarcopenia screening in patients undergoing CABG.

**Methods:**

A total of 2810 patients who underwent CABG were analysed and classified according to presence of reduced muscle mass. The skeletal muscle index (SMI) was calculated as L3 muscle area (cm^2^)/height (m)^2^ on computed tomography. Reduced SMI was defined as SMI ≤ 45 cm^2^/m^2^ in male and ≤ 38 cm^2^/m^2^ in female. The primary outcome was all‐cause mortality, and survival analysis was performed using the Kaplan–Meier method and compared with the log‐rank test.

**Results:**

The median follow‐up was 8.7 years, and 924 patients (32.9%) had reduced SMI. Patients with reduced SMI were older (67.7 ± 8.8 vs. 62.2 ± 9.8 years; *p* < 0.001) and less frequently male (69.8% vs. 81.1%; *p* < 0.001). SMI was significantly associated with risk of death on a restricted cubic spline curve (HR = 1.04 per‐1 decrease; 95% CI 1.03–1.05; *p* < 0.001). Patients with reduced SMI had a higher incidence of long‐term mortality than those with preserved SMI (survival rate 41.4% vs. 62.8%; HR_adj_ = 1.18, 95% CI 1.03–1.36, *p* = 0.020). Subgroup analysis showed that the prognostic implication of reduced SMI on long‐term survival was more evident in male (HR_adj_ = 2.01, 95% CI 1.72–2.35) than female (HR_adj_ = 1.28, 95% CI 0.98–1.68) (interaction *p* = 0.006).

**Conclusions:**

Reduced muscle mass, defined by SMI on computed tomography, was associated with long‐term mortality after CABG. These results provide contemporary data to allow the evaluation of physical frailty in patients with advanced coronary artery disease before surgery.

**Trial Registration:** Long‐Term Outcomes and Prognostic Factors in Patients Undergoing CABG or PCI: NCT03870815

## 1 Introduction

Coronary artery bypass grafting (CABG) is a safe and effective treatment option for patients with complex coronary artery disease (CAD), such as left main disease or significant stenosis in three major coronary arteries, who are not candidates for percutaneous coronary intervention [1, 2]. The prognosis of CABG has improved over the decades because of the evolution of surgery techniques and risk stratification [3, 4, 5]. Perioperative risk scoring systems, such as the European System for Cardiac Operative Risk Evaluation (EuroSCORE) II and the Society of Thoracic Surgeons (STS) risk score, have been applied to patients who are planning cardiovascular surgery in contemporary practice [6, 7]. Although poor mobility (subjective severe impairment of mobility secondary to musculoskeletal or neurological dysfunction) is included in EuroSCORE II, there remains a lack of a dedicated risk‐scoring system for addressing frailty and long‐term mortality in patients undergoing open heart surgery, including CABG. This gap is particularly critical for this population, as frailty poses a significant barrier to recovery and the ability to withstand the physiological stress of surgery.

Sarcopenia is characterized by generalized loss of skeletal muscle mass and function [8]. There are numerous proposed methods for defining sarcopenia and the most commonly used ones are based on the skeletal muscle index (SMI), which is obtained using dual energy X‐ray absorptiometry anthropometry or computed tomography (CT) scans [9]. Sarcopenia is a key determinant of patient vulnerability to adverse outcomes of various diseases such as sepsis, liver disease, cancer and heart failure [10, 11, 12, 13, 14, 15]. Additionally, sarcopenia may be an indicator of subclinical atherosclerosis and CAD [15, 16]. Because sarcopenia is associated with aging, its clinical implication might be informative owing to the present aged society with extended life expectancy. In routine clinical practice, physicians consistently evaluate the performance status of patients before initiating treatment. However, the physical status of patients planning to undergo CABG, an invasive and potentially life‐threatening surgery, is often not adequately assessed beforehand. Furthermore, there is a paucity of information regarding the relationship between sarcopenia screening and surgical outcomes. A previous study tried to elucidate the prognostic implication of preoperative sarcopenia screening, which was defined by psoas muscle area using CT scans, on postoperative outcomes [17]. Although that study showed that reduced muscle mass was associated with an increased mortality risk after CABG, it was limited by the small sample size and the heterogeneity of the time interval between CT scan and surgery.

Therefore, we sought to evaluate the long‐term prognostic implications of sarcopenia screening in patients with advanced CAD and undergoing CABG.

## 2 Methods

### 2.1 Study Population

**FIGURE 1:**
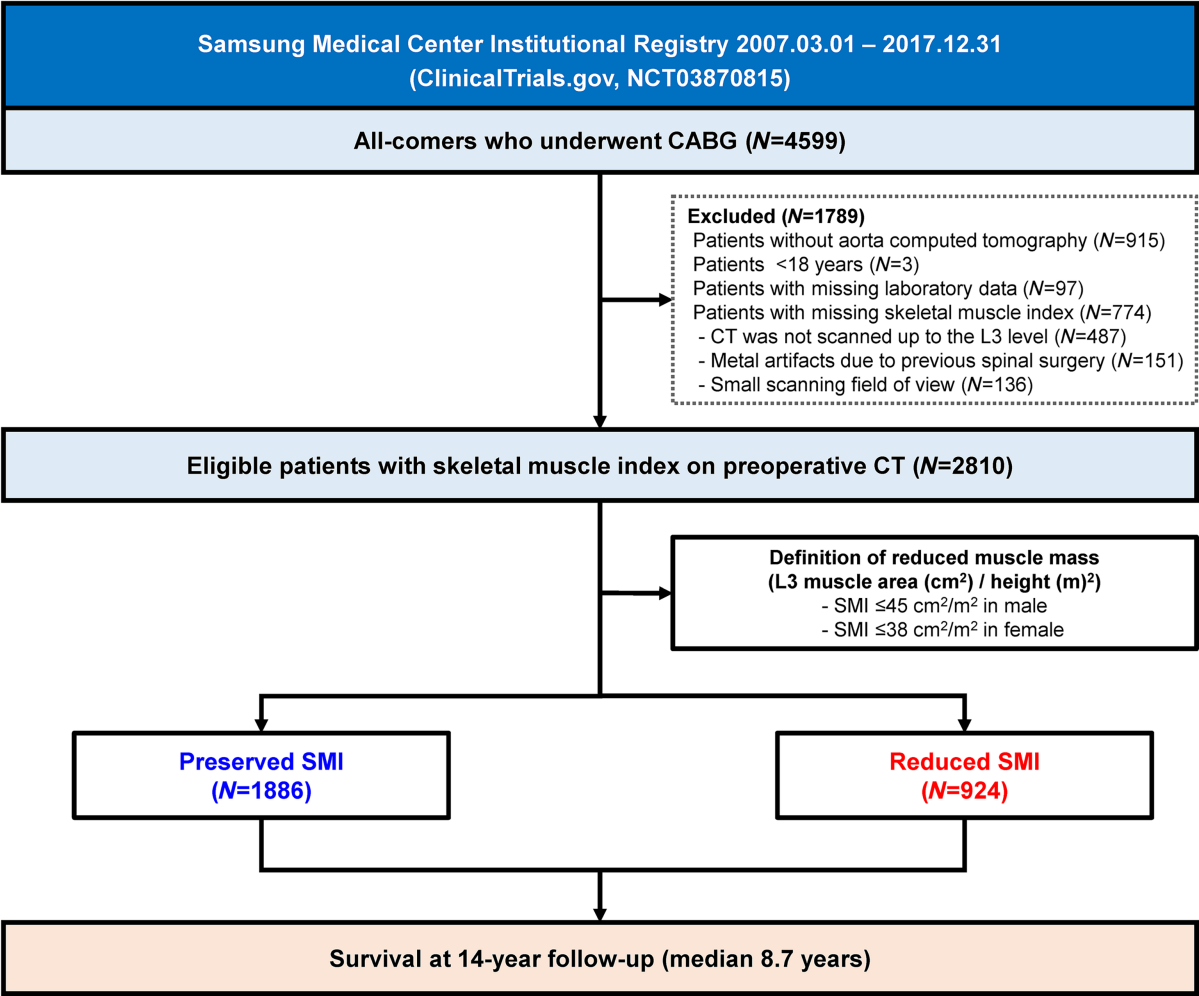
Study flow. CABG = coronary artery bypass grafting; CT = computed tomography; SMI = skeletal muscle index.

The study population was derived from the Samsung Medical Center institutional registry for patients undergoing CABG (ClinicalTrials.gov NCT03870815), and 4599 consecutive CAD patients who underwent CABG from 1 March 2007 to 31 December 2017 were enrolled in this analysis. Patients who did not undergo preoperative CT scans (*N* = 915), were under 18 years (*N* = 3), had missing laboratory data (*N* = 97) or had missing SMI data (*N* = 774; 487 patients were not scanned up to the L3 level, 151 patients had metal artefacts due to previous spinal surgery and 136 patients had a small scanning field of view for muscle quantification) were excluded from this study. Finally, a total of 2810 patients was included and stratified according to the presence of reduced SMI (Figure 1). This study protocol was approved and the requirement for informed consent from individual patients was waived by the Institutional Review Board of Samsung Medical Center.

### 2.2 SMI and Definition of Reduced Muscle Mass

Non‐contrast aorta CT scans were performed routinely as a preoperative evaluation for calcific disease of the aorta, internal carotid arteries, internal thoracic arteries and anatomical structure of the mediastinum. Skeletal muscle area was measured based on the cross‐sectional image at the level of the third lumbar vertebra using commercial software (Terarecon Intuition, version 4.4.11, San Matteo, CA, USA). The CT Hounsfield unit (HU) range used for specific tissue was −29 to 150 HU for skeletal muscle [18]. After applying threshold methods using a predefined HU threshold set for the image, boundaries between tissues were corrected manually when necessary. Dr. SM Kim was the chief of CT core lab and responsible for analyses, and the results were cross‐checked by two independent observers. SMI (cm^2^/m^2^) was calculated as skeletal muscle area (cm^2^)/height (m^2^) (Figure 1). Cut‐off values of the SMI for defining reduced muscle mass were 45 cm^2^/m^2^ for male and 38 cm^2^/m^2^ for female based on receiver‐operating characteristic (ROC) curve analysis for predicting all‐cause death during the follow‐up period (Figure S1).

### 2.3 Surgical Techniques

CABG was performed in accordance with relevant standard guidelines [19, 20]. Off‐pump CABG using the bilateral internal thoracic artery is the preferred technique at our institution. The adoption of perioperative treatment strategies, including the use of cardiopulmonary bypass, the number of grafts used, and the administration of concomitant medications after CABG, were all left to the operator's preference. Our current institutional guidelines instruct that all patients receive optimal medical treatment, including antiplatelet therapy (aspirin or P2Y_12_ inhibitor), beta‐blockers, or renin–angiotensin–aldosterone system blockers, if no contraindication exists [21, 22].

### 2.4 Outcomes Measurements and Follow‐Up

The primary outcome of the current study was all‐cause death at the 14‐year follow‐up (median 8.7 years) after surgery. Secondary outcomes were major adverse cardiac and cerebrovascular events (MACCE), a composite of all‐cause death, myocardial infarction (MI) and stroke. All deaths were considered cardiac unless a definite non‐cardiac cause was established. MI was defined based on elevated cardiac troponin or myocardial band fraction of creatine kinase greater than the upper reference limit with concomitant ischemic symptoms or electrocardiography findings indicative of ischemia based on the fourth universal definition of MI [23]. Stroke was defined as an episode of neurological dysfunction caused by vascular lesions of the brain such as haemorrhage, embolism, thrombosis, or rupturing aneurysm based on imaging or neuropathological evidence [24]. Mortality data for patients who were lost to follow‐up were confirmed by a review of the National Death Records. All events were adjudicated by an expert in cardiology who was blinded to the treatment strategy used.

### 2.5 Statistical Analysis

A detailed description of the statistical analysis is presented in the Supplementary Appendix. The optimal cut‐off value of the SMI for predicting 14‐year all‐cause death after CABG was analysed using ROC curves and the Youden method [25]. The Kaplan–Meier method was used for survival analysis and comparisons were made with the log‐rank test. Survival rates were computed for each component of the outcomes at 14 years after CABG. Cox proportional hazards regression models were constructed to calculate hazard ratios (HRs) and 95% confidence intervals (CIs). Proportional hazards assumptions for the models were assessed using Schoenfeld residuals. A multivariable Cox model was fitted using all variables with *p* < 0.1 from the univariable analyses and variables that could be clinically relevant. The selection of the optimal model was based on the Akaike information criterion (Tables S1 and S2).

As sensitivity analyses, propensity score matching (PSM) and inverse probability treatment weighting (IPTW) analysis were performed using the propensity score from a multivariable logistic regression model with a good balance (Table S3). Patients with reduced SMI were matched 1:1 with patients with preserved SMI via ‘nearest‐neighbour matching’ (a greedy match) without replacement and a calliper size predefined as 0.2. IPTW analyses were based on propensity scores. Additionally, we performed Bayesian modelling to assess the effect of unmeasured confounders on the summary estimates [26]. The posterior distribution of the model parameters was obtained using Markov‐chain Monte Carlo Gibbs sampling. The prior settings were defaulted to a mean of 0 and a variance of 106 for each variable, followed by 2000 burn‐in iterations and an additional 10 000 iterations. HRs and 95% credible intervals were calculated with Cox regression through Bayesian analysis. Credible intervals of the HRs that did not include 1 were considered significant. All analyses were two‐tailed, and statistical significance was defined as a *p* value less than 0.05. All statistical analyses were conducted using SPSS Statistics version 25.0 (IBM Corp., Armonk, NY, USA) and R version 4.2.3 (R Foundation for Statistical Computing, Vienna, Austria).

## 3 Results

### 3.1 Baseline Characteristics According to SMI

Table 1 summarizes the baseline characteristics of the study population according to SMI. Overall, 924 patients (32.9%) had reduced SMI in the preoperative CT assessment. Patients with reduced SMI were older (67.7 ± 8.8 vs. 62.2 ± 9.8 years; *p* < 0.001), and there was a lower prevalence of male than among patients with preserved SMI (69.8% vs. 81.1%; *p* < 0.001). Patients with reduced SMI weighed less (60.1 ± 9.4 vs. 68.7 ± 10.3 kg; *p* < 0.001) and had a lower body mass index (22.6 ± 2.6 vs. 25.5 ± 2.7  kg/m^2^; *p* < 0.001). Patients with reduced SMI showed lower visceral subcutaneous fat area than patients with preserved SMI. Conversely, intramuscular fat area was higher among the patients with reduced SMI than those with preserved SMI. The SMI was positively correlated with visceral fat index (normalized for height, same as SMI) (*R* = 0.16, *p* < 0.001), whereas it was negatively correlated with subcutaneous (*R* = −0.07, *p* < 0.001) and intramuscular fat indexes (*R* = −0.13, *p* < 0.001) (Figure S2).

**TABLE 1:** Baseline characteristics of the study population.

	Overall population (*N* = 2810)	Preserved SMI (*N* = 1886)	Reduced SMI (*N* = 924)	*p*
Age, years	64.0 ± 9.8	62.2 ± 9.8	67.7 ± 8.8	< 0.001
Male, *n* (%)	2174 (77.4)	1529 (81.1)	645 (69.8)	< 0.001
Height, cm	163.6 ± 8.3	164.0 ± 8.2	163.0 ± 8.5	0.003
Weight, kg	65.8 ± 10.8	68.7 ± 10.3	60.1 ± 9.4	< 0.001
Body mass index, kg/m^2^	24.5 ± 3.0	25.5 ± 2.7	22.6 ± 2.6	< 0.001
Skeletal muscle index, cm^2^/m^2^	46.9 ± 8.1	50.9 ± 6.2	38.6 ± 4.7	< 0.001
Skeletal muscle area, cm^2^	126.4 ± 27.6	137.7 ± 23.8	103.5 ± 19.5	< 0.001
Visceral fat area, cm^2^	160.1 ± 60.9	167.6 ± 60.5	144.7 ± 58.8	< 0.001
Subcutaneous fat area, cm^2^	135.7 ± 54.4	141.0 ± 54.8	124.9 ± 51.9	< 0.001
Intramuscular fat area, cm^2^	11.2 ± 10.6	10.9 ± 12.3	11.7 ± 5.7	0.015
Presentation with AMI	492 (17.5)	310 (16.4)	182 (19.7)	0.037
Cardiovascular risk factors				
Hypertension	1823 (64.9)	1231 (65.3)	592 (64.1)	0.059
Diabetes mellitus	1312 (46.7)	872 (46.2)	440 (47.6)	0.515
Dyslipidaemia	999 (35.6)	715 (37.9)	284 (30.7)	< 0.001
Current smoking	981 (34.9)	709 (37.6)	272 (29.4)	< 0.001
Chronic kidney disease	210 (7.5)	45 (3.3)	128 (9.5)	< 0.001
Previous stroke	373 (13.3)	230 (12.2)	143 (15.5)	0.019
Peripheral artery disease	186 (6.6)	118 (6.3)	68 (7.4)	0.306
Previous myocardial infarction	220 (7.8)	155 (8.2)	65 (7.0)	0.306
Previous PCI	526 (18.7)	379 (20.1)	147 (15.9)	0.009
Previous CABG	33 (1.2)	26 (1.4)	7 (0.8)	0.212
Preoperative LVEF, %	55.1 ± 13.8	55.8 ± 13.2	53.6 ± 14.7	< 0.001
Laboratory findings				
WBC, × 10^3^/mm^3^	7.0 ± 2.0	7.0 ± 1.9	7.1 ± 2.3	0.578
Haemoglobin, g/dL	13.1 ± 1.8	13.3 ± 1.8	12.6 ± 1.7	< 0.001
Creatinine, mg/dL	1.3 ± 1.4	1.3 ± 1.4	1.3 ± 1.4	0.952
Total cholesterol, mg/dL	158.3 ± 43.	159.3 ± 44.0	156.2 ± 42.9	0.079
LDL cholesterol, mg/dL	98.9 ± 39.1	100.0 ± 39.3	96.5 ± 38.8	0.036
HDL cholesterol, mg/dL	43.0 ± 11.5	42.2 ± 10.8	44.6 ± 12.7	< 0.001
hs‐CRP, mg/dL	0.8 ± 2.2	0.7 ± 1.8	1.1 ± 2.8	< 0.001

*Note:* Values are expressed as mean ± standard deviation or number (%).

Abbreviations: AMI = acute myocardial infarction; CABG = coronary artery bypass grafting; HDL = high‐density lipoprotein; hs‐CRP = high sensitivity C‐reactive protein; LDL = low‐density lipoprotein; LVEF = left ventricular ejection fraction; PCI = percutaneous coronary intervention; SMI = skeletal muscle index; WBC = white blood count.

Among traditional cardiovascular risk factors, patients with reduced SMI showed significantly higher rates of chronic kidney disease (9.5% vs. 3.3%; *p* < 0.0001) and previous stroke (15.5% vs. 12.2%; *p* = 0.019), whereas rates of dyslipidaemia (30.7% vs. 37.9%; *p* < 0.001), current smoking (29.4% vs. 37.6%; *p* < 0.001) and previous percutaneous coronary intervention (15.9% vs. 20.1%; *p* = 0.009) were lower compared with patients with preserved SMI. In laboratory studies, patients with reduced SMI had lower haemoglobin levels (12.6 ± 1.7 vs. 13.3 ± 1.8 g/dL; *p* < 0.001) and higher levels of high‐sensitivity C‐reactive protein (1.1 ± 2.8 vs. 0.7 ± 1.8 mg/dL; *p* < 0.001) than patients with preserved SMI.

### 3.2 Procedural Findings, Medical Management and In‐Hospital Outcomes

Table 2 summarizes procedural findings, medications and in‐hospital events. Patients with reduced SMI showed a higher incidence of three‐vessel disease (74.4% vs. 70.6%; *p* = 0.037) and underwent off‐pump CABG less frequently than patients with preserved SMI (79.5% vs. 85.0%; *p* < 0.001). There was no significant difference in bilateral internal thoracic artery grafting and saphenous vein grafting. After the index surgery, there was no significant difference in the rates of postoperative cardiopulmonary resuscitation, reoperation for bleeding or graft problems. Among maintenance treatments, lipid‐lowering therapy was less frequently prescribed for patients with reduced SMI than those with preserved SMI (76.4% vs. 82.6%; *p* < 0.001). There was no significant difference in the rate of in‐hospital death (1.4% in patients with reduced SMI vs. 0.7% in patients with preserved SMI; *p* = 0.135).

**TABLE 2:** Procedural characteristics, medications and in‐hospital outcomes.

	Overall population (*N* = 2810)	Preserved SMI (*N* = 1886)	Reduced SMI (*N* = 924)	*p*
Angiographic findings				
Multivessel disease	2639 (94.3)	1763 (93.8)	876 (95.3)	0.129
LM disease	598 (21.4)	403 (21.5)	195 (21.2)	0.923
One‐vessel disease	157 (5.6)	115 (6.1)	42 (4.6)	0.113
Two‐vessel disease	629 (22.5)	437 (23.3)	192 (20.9)	0.174
Three‐vessel disease	2010 (71.8)	1326 (70.6)	684 (74.4)	0.037
Procedure findings				
Off‐pump CABG	2338 (83.2)	1604 (85.0)	734 (79.5)	< 0.001
Number of anastomoses	4.0 ± 1.3	4.0 ± 1.3	4.1 ± 1.3	0.301
Bilateral internal thoracic artery grafting	2377 (85.1)	1595 (85.0)	782 (85.2)	0.954
Saphenous vein grafting	535 (19.1)	349 (18.6)	186 (20.2)	0.320
Postoperative CPR	28 (1.0)	14 (0.7)	14 (1.5)	0.082
Reoperation for bleeding	47 (1.7)	30 (1.6)	17 (1.8)	0.741
Reoperation for graft problem	5 (0.2)	3 (0.2)	2 (0.2)	0.666
Medications				
Dual antiplatelet therapy	2215 (78.8)	1475 (78.2)	740 (80.1)	0.273
RAAS blockade	638 (22.7)	438 (23.2)	200 (21.6)	0.373
Beta‐blockers	2229 (79.3)	1581 (83.8)	756 (81.8)	0.199
Lipid‐lowering therapy	2264 (80.6)	1558 (82.6)	706 (76.4)	< 0.001
Diuretics	2075 (73.8)	1402 (74.3)	673 (72.8)	0.421
In‐hospital death	27 (1.0)	14 (0.7)	13 (1.4)	0.135

*Note:* Values are expressed as mean ± standard deviation or number (%).

Abbreviations: CABG = coronary artery bypass grafting; CPR = cardiopulmonary resuscitation; LM = left main; RAAS = renin–angiotensin–aldosterone system; SMI = skeletal muscle index.

### 3.3 Clinical Outcomes After CABG According to SMI During Long‐Term Follow‐Up

**FIGURE 2:**
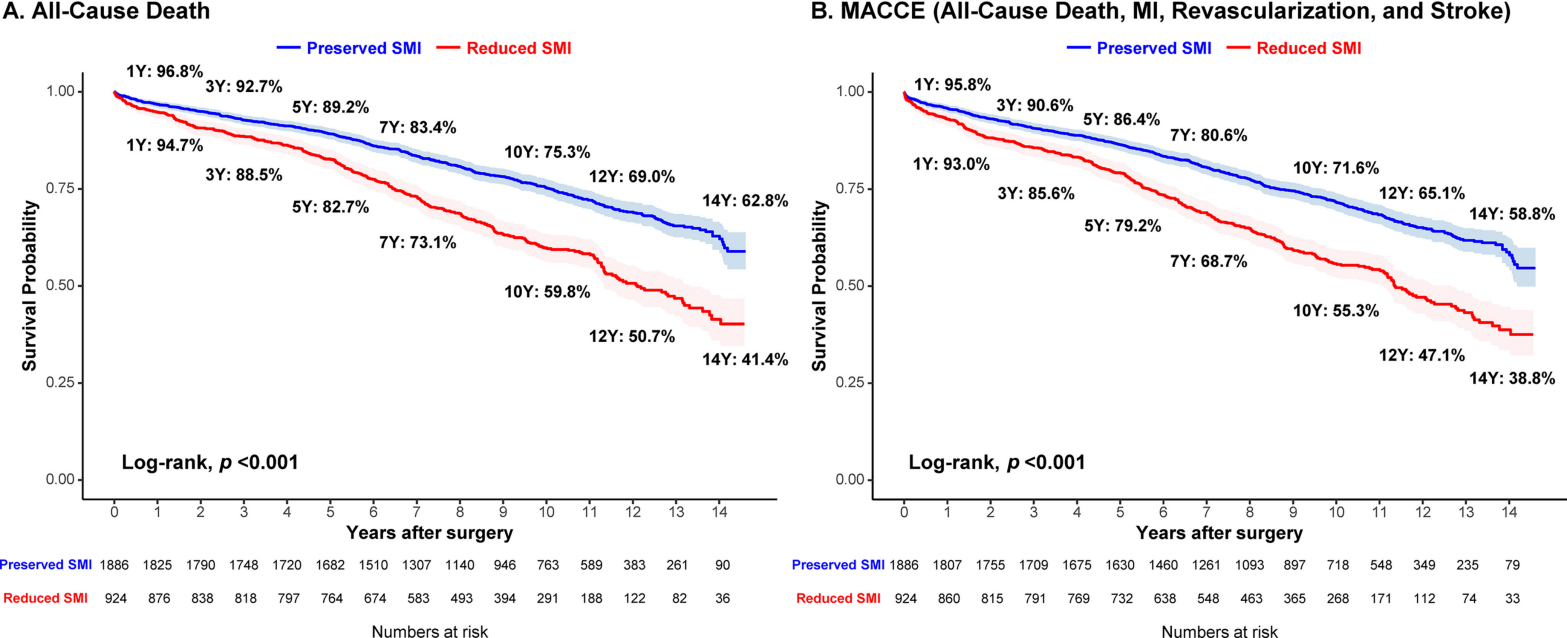
Kaplan–Meier curve for clinical outcomes according to SMI. The survival probability of (A) all‐cause death and (B) MACCE were compared according to the presence reduced SMI. MACCE = major adverse cardiac and cerebrovascular event.

The SMI was significantly associated with long‐term all‐cause death risk on a restricted cubic spline curve (HR 1.04 per‐1 decrease, 95% CI 1.03–1.05, *p* < 0.001) (Figure S3). Patients with reduced SMI had a higher incidence of long‐term mortality than those with preserved SMI (survival rate 41.4% vs. 62.8%; adjusted HR 1.18, 95% CI 1.03–1.36, *p* = 0.020). The MACCE‐free survival rate was also significantly lower in the patients with reduced SMI than in the patients with preserved SMI (survival rate 38.8% vs. 58.8%; adjusted HR 1.24, 95% CI 1.09–1.42, *p* = 0.001) (Figure 2, Table 3, Table S4). In sensitivity analysis, the results were consistent with reduced SMI association with increased risk in all‐cause death (PSM‐adjusted HR 1.23, 95% CI 1.06–1.42, *p* = 0.008; and IPTW‐adjusted HR 1.24, 95% CI 1.07–1.43, *p* = 0.005) and MACCE (PSM‐adjusted HR 1.28, 95% CI 1.11–1.48, *p* < 0.001; and IPTW‐adjusted HR 1.29, 95% CI 1.12–1.49, *p* < 0.001) (Table 3). The Bayesian proportional hazards modelling for unmeasured confounders also demonstrated consistent trends as in the original analysis.

**TABLE 3:** Clinical outcomes according to reduced SMI during a median 8.7‐year follow‐up.

	Multivariable adjusted,^a^ HR (95% CI)	*p*	PSM‐adjusted, HR (95% CI)	*p*	IPTW‐adjusted, HR (95% CI)	*p*	Bayesian PH (95% CrI)
All‐cause death	1.18 (1.03–1.36)	0.020	1.23 (1.06–1.42)	0.008	1.24 (1.07–1.43)	0.005	1.25 (1.08–1.42)
Myocardial infarction	1.96 (0.98–3.90)	0.056	1.81 (0.80–4.09)	0.156	2.06 (1.00–4.21)	0.049	2.01 (0.97–3.68)
Any revascularization	1.74 (1.04–2.93)	0.037	1.39 (0.77–2.50)	0.280	1.71 (1.00–2.94)	0.052	1.18 (0.75–1.76)
Stroke	1.20 (0.79–1.81)	0.386	1.26 (0.78–2.03)	0.337	1.27 (0.83–1.95)	0.271	1.62 (0.88–2.73)
MACCE	1.24 (1.09–1.42)	0.001	1.28 (1.11–1.48)	< 0.001	1.29 (1.12–1.49)	< 0.001	1.28 (1.11–1.45)

Abbreviations: CI = confidence interval; CrI = credible interval; HR = hazard ratio; IPTW = inverse probability treatment weighting; MACCE = major adverse cardiac and cerebrovascular event; PH = proportional hazards; PSM = propensity score matching PH, proportional hazards; SMI = skeletal muscle index.

^a^ Adjusted for variables of age, sex, presentation with acute myocardial infarction, hypertension, dyslipidaemia, current smoking, chronic kidney disease, previous stroke, previous PCI, preoperative left ventricular ejection fraction, three‐vessel disease, off‐pump coronary artery bypass grafting and lipid‐lowering therapy.

**FIGURE 3:**
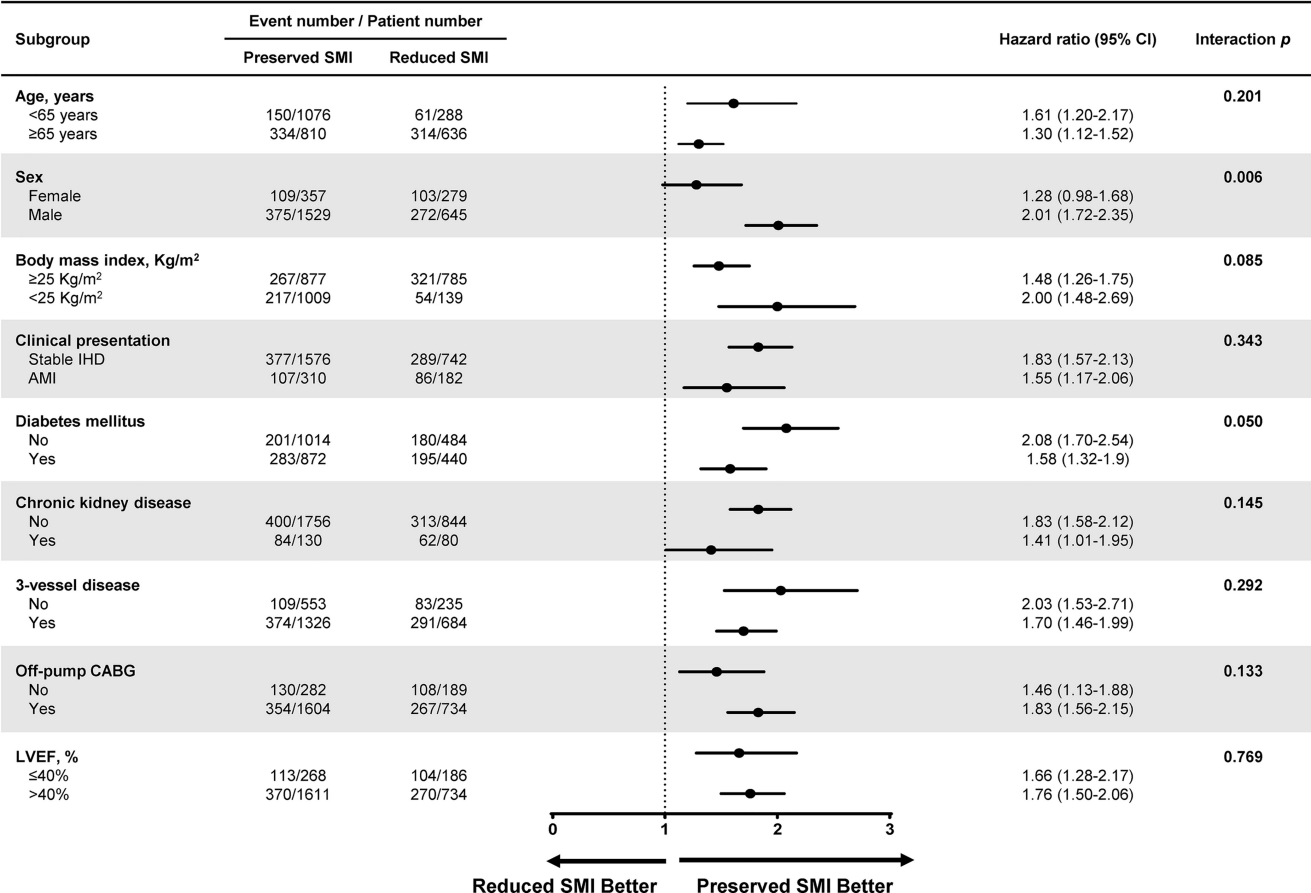
Subgroup analysis of death during a median 8.7‐year follow‐up. Subgroup analysis demonstrated a differential prognostic impact of reduced SMI between male and female. AMI = acute myocardial infarction; CABG = coronary artery bypass grafting; CI = confidence interval; IHD = ischemic heart disease; LVEF = left ventricular ejection fraction.

Subgroup analysis showed that the prognostic implication of reduced SMI was consistent across the subgroups in age (< 65 vs. ≥ 65 years), body mass index (≥ 25 kg/m^2^ vs. < 25 kg/m^2^), clinical presentation (stable ischemic heart disease vs. acute MI), diabetes mellitus, chronic kidney disease, three‐vessel disease, off‐pump CABG and left ventricular ejection fraction (≤ 40% vs. > 40%) (Figure 3). The prognostic implication of reduced SMI for long‐term survival was more evident among male (adjusted HR 2.01, 95% CI 1.72–2.35) than female (adjusted HR 1.28, 95% CI 0.98–1.68) (interaction *p* = 0.006).

## 4 Discussion

Although preoperative sarcopenia is a surrogate marker of physical frailty and might be associated with clinical outcomes, the assessment of sarcopenia has not been a major consideration in contemporary practice for patients who are planning CABG. This study reported the optimal cut‐off values of SMI and the long‐term prognostic impact of sarcopenia screening as assessed by SMI on CT scans before CABG. The major findings are as follows. First, reduced muscle mass was not a rare condition in patients who underwent CABG, and about one‐third of patients had reduced SMI. Second, the patients with reduced SMI were older; more frequently female; and not related to traditional cardiovascular risk factors, such as hypertension, diabetes mellitus, dyslipidaemia or smoking. Third, SMI was negatively associated with all‐cause death risk. Reduced SMI was significantly associated with an increased risk of death and adverse cardiovascular outcomes during a median of 8.7 years after surgery, and these results were consistent after adjusting confounders by multivariable, PSM, and IPTW, and Bayesian analyses. The prognostic implication was more evident among male than female who underwent CABG.

Sarcopenia has been defined as age‐related loss of muscle mass and/or low muscle strength [27, 28, 29]. Traditionally, sarcopenia was considered part of the aging process in older patients; however, it is now recognized that sarcopenia can begin earlier in life [30]. According to consensus documents from Asian, European and American societies [27, 28, 29], various methods for quantifying and qualifying muscle have been recommended, including grip strength, dual‐energy X‐ray absorptiometry, bioelectrical impedance analysis and walking test for gait speed. As an alternative tool for quantifying skeletal muscle mass, identification of the psoas muscle at the L3 level on a CT scan is an emerging technique. This method has been highly reproducible and associated with prognosis in various clinical settings, such as sepsis, liver disease, cancer and heart failure [10, 11, 12, 13, 14, 15]. As with other critical illnesses, CABG is highly stressful for patients with decreased physical reserve, and the outcomes of patients with reduced muscle mass after surgery could be affected by underlying general medical conditions.

There is limited evidence that reduced muscle mass is associated with poor prognosis after CABG. Okamura et al. evaluated 304 patients who underwent off‐pump CABG and had preoperative abdominal CT data for assessing SMI within 3 months before surgery [17]. In that study, reduced muscle mass was defined as SMI below the 25^th^ sex‐specific percentile in the study population. Although reduced SMI was not associated with in‐hospital complications and mortality, reduced SMI was associated with an increased risk of death and adverse cardiovascular outcomes including cardiac death, MI, revascularization and stroke during a median follow‐up of 4.5 years. However, after multivariable adjustment, the prognostic implication of reduced SMI in terms of adverse cardiovascular outcomes was diminished. This study was limited in that the optimal cut‐off values for reduced SMI were ambiguous, and the time interval between CT and surgery was heterogeneous. In the current study, a large sample size of consecutive patients who underwent CABG and preoperative CT scans was analysed, and a clear association between SMI and long‐term outcomes was demonstrated. Notably, the cut‐off values for reduced SMI were determined considering sex difference (SMI ≤ 45 cm^2^/m^2^ in male and ≤ 38 cm^2^/m^2^ in female) based on optimal values for predicting future clinical events. Considering previous imaging studies of cancer patients, which defined reduced muscle mass as SMI 33–55 cm^2^/m^2^ for male and SMI 29–42 cm^2^/m^2^ for female [31], these cut‐offs for reduced SMI are consistent and acceptable. Approximately 33% of patients (29.7% in male and 43.9% in female) were stratified to patients with reduced SMI, and reduced SMI was an independent predictor for all‐cause mortality and adverse cardiovascular outcomes during long‐term follow‐up.

Patients with reduced SMI and CAD are older with low body weight and body mass index. Interestingly, patients with reduced SMI had a similar incidence of traditional cardiovascular risk factors compared with patients with preserved SMI, even though there was lower incidence of dyslipidaemia and smoking. Our study suggests that patients with reduced SMI are at risk of chronic poor nutrition, inflammation, or metabolic consumption, which can be explained by low lipid levels and high levels of inflammatory markers. Because sarcopenia has been linked to increased inflammation and oxidative stress, both of which are known contributors to atherosclerosis [32], sarcopenia may be related to subclinical atherosclerosis and CAD [15, 16]. Furthermore, there may be sex differences in the association between reduced SMI and long‐term clinical outcomes based on subgroup analysis. Previous studies have also shown that two‐thirds of frail male with cirrhosis had sarcopenia, whereas only one‐quarter of frail female had sarcopenia, and there was a stronger association between sarcopenia and mortality in male [33, 34]. Therefore, diagnosis and management of reduced muscle mass are essential for patients with CAD, particularly in male, even after successful revascularization with CABG during long‐term follow‐up.

### 4.1 Perspectives

Given the limitations of traditional scoring systems, our study demonstrates that preoperative assessment of sarcopenia screening using CT can be a useful method for assessing physical frailty and predicting risk of long‐term mortality following CABG. Although future studies are needed to confirm the clinical benefit of addressing sarcopenia screening during the perioperative period, our findings suggest that prognostication and management of reduced muscle mass, even after surgery, may improve outcomes.

### 4.2 Study Limitations

This study has some limitations that should be considered. First, it was a single‐center retrospective study that might have unexpected confounding factors, including selection bias. However, because Samsung Medical Center is a highly experienced and high‐volume centre for CABG in Korea, patients who need CABG are from a nationwide population; therefore, a large sample size could be enabled for the present analysis. Although we performed sensitivity analyses to adjust for confounders as much as possible, some selection bias might have remained. The measured or unmeasured confounders were adjusted to minimize the bias from different baseline clinical profiles between patients with reduced and preserved SMI. Second, our routine clinical practice after surgery is that patients are referred back to local clinics after stabilization. Although there remains the risk of under‐reporting clinical events, the mortality data were provided by National Death Records, which guarantees their accuracy. Third, the medical cost of CT is covered by the National Health Insurance Service in Korea; therefore, cost‐effectiveness is not a documented issue among patients and clinicians. However, the cost‐effectiveness of CT for evaluating sarcopenia screening might be challenging in other countries with different medical systems. Fourth, identifying skeletal muscle area at the L3 level on CT is a simple method with high reproducibility. However, it only reflects the muscle mass and might be limited when assessing muscle strength or function, which is related to patient performance. In addition, the use of the same cohort to define cut‐offs for reduced muscle mass and to assess mortality is a significant limitation of the current study, and the findings should be validated in further research.

## 5 Conclusions

Although managing traditional cardiovascular risk factors is an imperative option for a better long‐term prognosis, sarcopenia, a surrogate marker of physical frailty, is also a challenging issue in aging and aged populations. Nevertheless, sarcopenia screening has been underestimated in contemporary practice, and traditional risk scoring systems could not fully reflect the frailty of the patients. This study finds that reduced muscle mass can be easily defined by SMI on perioperative CT scans and is associated with long‐term clinical outcomes after CABG for patients with CAD.

## Conflicts of Interest

The authors declare no conflicts of interest.

## Supporting information


**Table S1.** Predictors of All‐Cause Death After Coronary Artery Bypass Grafting During a Median 8.7‐Year Follow‐Up
**Table S2.** Predictors of MACCE After Coronary Artery Bypass Grafting During a Median 8.7‐year Follow‐up
**Table S3.** Baseline Characteristics of the PSM‐Matched Population
**Table S4.** Comparison of Clinical Outcomes According to Reduced SMI at 14 Years (Median 8.7‐Year Follow‐Up)
**Figure S1.** Determination of the Cut‐Off Value of the Skeletal Muscle Index for Predicting Death
**Figure S2.** Relationship between Skeletal Muscle Index and Fat
**Figure S3.** Predicting All‐Cause Death with Skeletal Muscle Index as a Continuous Variable During a Median 8.7‐Year Follow‐Up

## Data Availability

Original data used in the current analysis will be shared upon reasonable request. Any relevant inquiry should be emailed to Dr. Jeong Hoon Yang (Email: jhysmc@gmail.com).

## References

[1] S. J. Head , T. M. Kieser , V. Falk , H. A. Huysmans , and A. P. Kappetein , “Coronary Artery Bypass Grafting: Part 1—The Evolution Over the First 50 Years,” European Heart Journal 34 (2013): 2862–2872. 24086085 10.1093/eurheartj/eht330

[2] J. S. Lawton , J. E. Tamis‐Holland , S. Bangalore , et al., “2021 ACC/AHA/SCAI Guideline for Coronary Artery Revascularization: A Report of the American College of Cardiology/American Heart Association Joint Committee on Clinical Practice Guidelines,” Journal of the American College of Cardiology 79 (2022): e21–e129. 34895950 10.1016/j.jacc.2021.09.006

[3] A. Pu , L. Ding , J. Shin , et al., “Long‐Term Outcomes of Multiple Arterial Coronary Artery Bypass Grafting: A Population‐Based Study of Patients in British Columbia, Canada,” JAMA Cardiology 2 (2017): 1187–1196. 29049458 10.1001/jamacardio.2017.3705 PMC5710366

[4] C. McNeely , S. Markwell , and C. Vassileva , “Trends in Patient Characteristics and Outcomes of Coronary Artery Bypass Grafting in the 2000 to 2012 Medicare Population,” The Annals of Thoracic Surgery 102 (2016): 132–138. 26941075 10.1016/j.athoracsur.2016.01.016

[5] S. C. Hardiman , Y. F. Villan Villan , J. M. Conway , K. J. Sheehan , and B. Sobolev , “Factors Affecting Mortality After Coronary Bypass Surgery: A Scoping Review,” Journal of Cardiothoracic Surgery 17 (2022): 45. 35313895 10.1186/s13019-022-01784-z PMC8935749

[6] S. A. Nashef , F. Roques , L. D. Sharples , et al., “EuroSCORE II,” European Journal of Cardio‐Thoracic Surgery 41 (2012): 734–744 discussion 44–5. 22378855 10.1093/ejcts/ezs043

[7] S. M. O'Brien , L. Feng , X. He , et al., “The Society of Thoracic Surgeons 2018 Adult Cardiac Surgery Risk Models: Part 2‐Statistical Methods and Results,” The Annals of Thoracic Surgery 105 (2018): 1419–1428. 29577924 10.1016/j.athoracsur.2018.03.003

[8] A. J. Cruz‐Jentoft and J. P. Michel , “Sarcopenia: A Useful Paradigm for Physical Frailty,” European Geriatric Medicine. 4 (2013): 102–105.

[9] T. N. Kim and K. M. Choi , “Sarcopenia: Definition, Epidemiology, and Pathophysiology,” Journal of Bone Metabolism 20 (2013): 1–10. 24524049 10.11005/jbm.2013.20.1.1 PMC3780834

[10] D. Kim , K. Wijarnpreecha , K. K. Sandhu , G. Cholankeril , and A. Ahmed , “Sarcopenia in Nonalcoholic Fatty Liver Disease and All‐Cause and Cause‐Specific Mortality in the United States,” Liver International 41 (2021): 1832–1840. 33641244 10.1111/liv.14852

[11] P. C. Au , H. L. Li , G. K. Lee , et al., “Sarcopenia and Mortality in Cancer: A Meta‐Analysis,” Osteoporos Sarcopenia. 7 (2021): S28–s33. 33997306 10.1016/j.afos.2021.03.002 PMC8088991

[12] H. J. Oh , J. H. Kim , H. R. Kim , et al., “The Impact of Sarcopenia on Short‐Term and Long‐Term Mortality in Patients With Septic Shock,” Journal of Cachexia, Sarcopenia and Muscle 13 (2022): 2054–2063. 35478354 10.1002/jcsm.12995 PMC9397556

[13] J. Xu , C. S. Wan , K. Ktoris , E. M. Reijnierse , and A. B. Maier , “Sarcopenia Is Associated With Mortality in Adults: A Systematic Review and Meta‐Analysis,” Gerontology 68 (2022): 361–376. 34315158 10.1159/000517099

[14] Q. Xue , J. Wu , Y. Ren , J. Hu , K. Yang , and J. Cao , “Sarcopenia Predicts Adverse Outcomes in an Elderly Population With Coronary Artery Disease: A Systematic Review and Meta‐Analysis,” BMC Geriatrics 21 (2021): 493. 34521369 10.1186/s12877-021-02438-w PMC8439080

[15] A. Bielecka‐Dabrowa , N. Ebner , M. R. Dos Santos , J. Ishida , G. Hasenfuss , and S. von Haehling , “Cachexia, Muscle Wasting, and Frailty in Cardiovascular Disease,” European Journal of Heart Failure 22 (2020): 2314–2326. 32949422 10.1002/ejhf.2011

[16] J. E. Jun , M. Kang , S. M. Jin , et al., “Additive Effect of Low Skeletal Muscle Mass and Abdominal Obesity on Coronary Artery Calcification,” European Journal of Endocrinology 184 (2021): 867–877. 33852417 10.1530/EJE-20-0885

[17] H. Okamura , N. Kimura , M. Mieno , K. Yuri , and A. Yamaguchi , “Preoperative Sarcopenia Is Associated With Late Mortality After Off‐Pump Coronary Artery Bypass Grafting,” European Journal of Cardio‐Thoracic Surgery 58 (2020): 121–129. 31995164 10.1093/ejcts/ezz378

[18] E. Y. Kim , Y. S. Kim , I. Park , H. K. Ahn , E. K. Cho , and Y. M. Jeong , “Prognostic Significance of CT‐Determined Sarcopenia in Patients With Small‐Cell Lung Cancer,” Journal of Thoracic Oncology 10 (2015): 1795–1799. 26484630 10.1097/JTO.0000000000000690

[19] L. D. Hillis , P. K. Smith , J. L. Anderson , et al., “2011 ACCF/AHA Guideline for Coronary Artery Bypass Graft Surgery: A Report of the American College of Cardiology Foundation/American Heart Association Task Force on Practice Guidelines,” Circulation 124 (2011): e652–e735. 22064599 10.1161/CIR.0b013e31823c074e

[20] F. J. Neumann , M. Sousa‐Uva , A. Ahlsson , et al., “2018 ESC/EACTS Guidelines on Myocardial Revascularization,” European Heart Journal 40 (2019): 87–165. 30615155 10.1093/eurheartj/ehy855

[21] M. Valgimigli , H. Bueno , R. A. Byrne , et al., “2017 ESC Focused Update on Dual Antiplatelet Therapy in Coronary Artery Disease Developed in Collaboration With EACTS: The Task Force for Dual Antiplatelet Therapy in Coronary Artery Disease of the European Society of Cardiology (ESC) and of the European Association for Cardio‐Thoracic Surgery (EACTS),” European Heart Journal 39 (2018): 213–260. 28886622 10.1093/eurheartj/ehx419

[22] G. N. Levine , E. R. Bates , J. A. Bittl , et al., “2016 ACC/AHA Guideline Focused Update on Duration of Dual Antiplatelet Therapy in Patients With Coronary Artery Disease: A Report of the American College of Cardiology/American Heart Association Task Force on Clinical Practice Guidelines,” Journal of the American College of Cardiology 68 (2016): 1082–1115. 27036919 10.1016/j.jacc.2016.03.512

[23] K. Thygesen , J. S. Alpert , A. S. Jaffe , et al., “Fourth Universal Definition of Myocardial Infarction (2018),” European Heart Journal 40 (2019): 237–269. 30165617 10.1093/eurheartj/ehy462

[24] R. L. Sacco , S. E. Kasner , J. P. Broderick , et al., “An Updated Definition of Stroke for the 21st Century: A Statement for Healthcare Professionals From the American Heart Association/American Stroke Association,” Stroke 44 (2013): 2064–2089. 23652265 10.1161/STR.0b013e318296aeca PMC11078537

[25] R. Fluss , D. Faraggi , and B. Reiser , “Estimation of the Youden Index and Its Associated Cutoff Point,” Biometrical Journal 47 (2005): 458–472. 16161804 10.1002/bimj.200410135

[26] L. C. McCandless , P. Gustafson , and A. Levy , “Bayesian Sensitivity Analysis for Unmeasured Confounding in Observational Studies,” Statistics in Medicine 26 (2007): 2331–2347. 16998821 10.1002/sim.2711

[27] S. Bhasin , T. G. Travison , T. M. Manini , et al., “Sarcopenia Definition: The Position Statements of the Sarcopenia Definition and Outcomes Consortium,” Journal of the American Geriatrics Society 68 (2020): 1410–1418. 32150289 10.1111/jgs.16372 PMC12132920

[28] L. K. Chen , J. Woo , P. Assantachai , et al., “Asian Working Group for Sarcopenia: 2019 Consensus Update on Sarcopenia Diagnosis and Treatment,” Journal of the American Medical Directors Association 21 (2020): 300–307.e2. 32033882 10.1016/j.jamda.2019.12.012

[29] A. J. Cruz‐Jentoft , G. Bahat , J. Bauer , et al., “Sarcopenia: Revised European Consensus on Definition and Diagnosis,” Age and Ageing 48 (2019): 16–31. 30312372 10.1093/ageing/afy169 PMC6322506

[30] A. A. Sayer , H. Syddall , H. Martin , H. Patel , D. Baylis , and C. Cooper , “The Developmental Origins of Sarcopenia,” The Journal of Nutrition, Health & Aging 12 (2008): 427–432. 10.1007/BF02982703 PMC2652119 18615224

[31] H. Su , J. Ruan , T. Chen , E. Lin , and L. Shi , “CT‐Assessed Sarcopenia Is a Predictive Factor for Both Long‐Term and Short‐Term Outcomes in Gastrointestinal Oncology Patients: A Systematic Review and Meta‐Analysis,” Cancer Imaging 19 (2019): 82. 31796090 10.1186/s40644-019-0270-0 PMC6892174

[32] S. J. Meng and L. J. Yu , “Oxidative Stress, Molecular Inflammation and Sarcopenia,” International Journal of Molecular Sciences 11 (2010): 1509–1526. 20480032 10.3390/ijms11041509 PMC2871128

[33] L. Fozouni , C. W. Wang , and J. C. Lai , “Sex Differences in the Association Between Frailty and Sarcopenia in Patients With Cirrhosis,” Clinical and Translational Gastroenterology 10 (2019): e00102. 31789932 10.14309/ctg.0000000000000102 PMC6970562

[34] H. Feng , X. Wang , L. Mao , et al., “Relationship Between Sarcopenia/Myosteatosis and Frailty in Hospitalized Patients With Cirrhosis: A Sex‐Stratified Analysis,” Therapeutic Advances in Chronic Disease 12 (2021): 20406223211026996. 34377386 10.1177/20406223211026996 PMC8320564

