# Supplementary material for: Clinical Impact of Sarcopenia Screening on Long‐Term Mortality in Patients Undergoing Coronary Bypass Grafting
Source: J Cachexia Sarcopenia Muscle. 2024 Nov 8;15(6):2842–51. doi: 10.1002/jcsm.13645 (PMC11634471; doi:10.1002/jcsm.13645)
Supplement: Supplementary file 1 — Table S1. Predictors of All‐Cause Death After Coronary Artery Bypass Grafting During a Median 8.7‐Year Follow‐Up Table S2. Predictors of MACCE After Coronary Artery Bypass Grafting During a Median 8.7‐year Follow‐up Table S3. Baseline Characteristics of the PSM‐Matched Population Table S4. Comparison of Clinical Outcomes According to Reduced SMI at 14 Years (Median 8.7‐Year Follow‐Up) Figure S1. Determination of the Cut‐Off Value of the Skeletal Muscle Index for Predicting Death Figure S2. Relationship between Skeletal Muscle Index and Fat Figure S3. Predicting All‐Cause Death with Skeletal Muscle Index as a Continuous Variable During a Median 8.7‐Year Follow‐Up [file JCSM-15-2842-s001.docx]

**SUPPLEMENTARY APPENDIX**

**Clinical Impact of Sarcopenia Screening on Long-term Mortality in Patients with Undergoing Coronary Bypass Grafting**

**Seung Hun Lee, MD, PhD^1†^, Jinhwan Jo, MD^2†^, Jeong Hoon Yang, MD, et al.**

- **Supplemental Methods**
- **Supplemental Tables**
- Supplementary Table 1. Predictors of All-Cause Death After Coronary Artery Bypass Grafting During a Median 8.7-Year Follow-Up
- Supplementary Table 2. Predictors of MACCE After Coronary Artery Bypass Grafting During a Median 8.7-year Follow-up
- Supplementary Table 3. Baseline Characteristics of the PSM-Matched Population
- Supplementary Table 4. Comparison of Clinical Outcomes According to Reduced SMI at 14 Years (Median 8.7-Year Follow-Up)
- **Supplemental Figures**
- Supplementary Figure 1. Determination of the Cut-Off Value of the Skeletal Muscle Index for Predicting Death
- Supplementary Figure 2. Relationship between Skeletal Muscle Index and Fat
- Supplementary Figure 3. Predicting All-Cause Death with Skeletal Muscle Index as a Continuous Variable During a Median 8.7-Year Follow-Up
- **References**

**Supplemental Methods**

Discrete or categorical variables were compared using the Chi-square test or Fisher’s exact test. Continuous variables were analyzed using unpaired *t*-tests or Mann–Whitney rank-sum tests depending on their distribution. For correlation analysis, Pearson coefficients were calculated. Restricted cubic spline curves with three knots were used to evaluate the continuous effects of skeletal muscle index (SMI) on all-cause mortality.[1]

Since there were significant differences in baseline characteristics, which could significantly affect prognosis, sensitivity analyses were performed to adjust for confounders as much as possible. Frist, a multivariable Cox model was fitted using all variables with P <0.1 from the univariable analyses and variables that could be clinically relevant. The selection of the optimal model was based on the Akaike information criterion (**eTables 1 and 2**). The final model included variables of age, sex, presentation with acute myocardial infarction, hypertension, dyslipidemia, current smoking, chronic kidney disease, previous stroke, previous PCI, preoperative left ventricular ejection fraction, three-vessel disease, off-pump coronary artery bypass grafting, lipid-lowering therapy. Second, propensity score-matching (PSM) and inverse probability treatment-weighting (IPTW) analysis were performed using the propensity score from a multivariable logistic regression model. Patients with reduced SMI were matched 1:1 with patients with preserved SMI via “nearest-neighbor matching” (a greedy match) without replacement and a caliper size predefined as 0.2. IPTW analyses were based on propensity scores. Residual differences in characteristics between matched cohorts were assessed by calculating the absolute standardized mean differences. Standardized mean differences were less than 0.1 across all matched covariates (age, sex, presentation with acute MI, hypertension, diabetes mellitus, dyslipidemia, smoking, previous stroke, previous percutaneous coronary intervention, and off-pump CABG), indicating a good balance (**eTable 3**). All CIs for the inverse probability weighting analyses were assessed with the bootstrapping methods with 1,000 iterations.[2] Third, since the aforementioned analyses can only adjust the effect of measured confounders between comparative groups, we conducted Bayesian modeling, incorporating internal validation data as an additional sensitivity analysis to assess the impact of unmeasured confounders on the summary estimates.[3] The posterior distribution of the model parameters was obtained using Markov-chain Monte Carlo Gibbs sampling. The prior settings were defaulted to a mean of 0 and a variance of 10^6^ for each variable, followed by 2,000 burn-in iterations and an additional 10,000 iterations. The Bayesian estimators were adjusted for unmeasured confounding by integrating both internal validation and main study data, as outlined previously.[3] The hazard ratios (HR) and 95% credible intervals were calculated as a result of Cox regression through Bayesian analysis. The credible intervals of the hazard ratios (HR), which did not include 1, were considered significant.

**Supplementary Table 1. Predictors of All-Cause Death After Coronary Artery Bypass Grafting During a Median 8.7-Year Follow-Up**

|  | **Univariable analysis** | | | **Multivariable analysis^*^** | |
| --- | --- | --- | --- | --- | --- |
|  | **Hazard ratio (95% CI)** | | ***P* value** | **Hazard ratio (95% CI)** | ***P* value** |
| **Reduced muscle mass**  - Skeletal Muscle index ≤45 cm^2^/m^2^ in male  - Skeletal Muscle index ≤38 cm^2^/m^2^ in female | **1.78 (1.56-2.04)** | **< 0.001** | | **1.18 (1.03-1.36)** | **0.020** |
| Age (every 1-year increase) | 1.08 (1.08-1.09) | < 0.001 | | 1.08 (1.07-1.09) | <0.001 |
| Men | 0.93 (0.79-1.08) | 0.340 | | 1.13 (0.95-1.33) | 0.157 |
| Presentation with AMI | 1.72 (1.47-2.02) | < 0.001 | | 1.16 (0.99-1.38) | 0.075 |
| Hypertension | 1.44 (1.24-1.67) | < 0.001 | | 1.11 (0.95-1.29) | 0.198 |
| Dyslipidemia | 0.75 (0.65-0.86) | < 0.001 | | 0.85 (0.73-0.98) | 0.029 |
| Current smoking | 0.89 (0.77-1.02) | 0.101 | | 1.15 (0.99-1.35) | 0.069 |
| Chronic kidney disease | 4.63 (3.86-5.54) | < 0.001 | | 3.24 (2.69-3.92) | <0.001 |
| Previous stroke | 1.91 (1.62-2.25) | < 0.001 | | 1.36 (1.15-1.62) | <0.001 |
| Previous PCI | 1.17 (0.99-1.38) | 0.067 | | 1.19 (1.00-1.41) | 0.047 |
| Preoperative LVEF (every 1% increase) | 0.97 (0.97-0.98) | < 0.001 | | 0.98 (0.98-0.99) | <0.001 |
| Three-vessel disease | 1.41 (1.2-1.65) | < 0.001 | | 1.25 (1.06-1.47) | 0.008 |
| Off-pump coronary artery bypass grafting | 0.44 (0.37-0.51) | < 0.001 | | 0.86 (0.73-1.02) | 0.075 |
| Lipid-lowering therapy | 0.45 (0.39-0.52) | < 0.001 | | 0.67 (0.58-0.78) | <0.001 |

The c-statistic of the multivariable model was 0.77.

^*^ Multivariable Cox regression model was constructed using variables with P <0.1 or clinical relevance.

Abbreviations: AMI = acute myocardial infarction; CI = confidence interval; LVEF = left ventricular ejection fraction; PCI = percutaneous coronary intervention.

**Supplementary Table 2. Predictors of MACCE After Coronary Artery Bypass Grafting During a Median 8.7-year Follow-up**

|  | **Univariable analysis** | | | **Multivariable analysis^*^** | |
| --- | --- | --- | --- | --- | --- |
|  | **Hazard ratio (95% CI)** | | ***P* value** | **Hazard ratio (95% CI)** | ***P* value** |
| **Reduced muscle mass**  - Skeletal Muscle index ≤45 cm^2^/m^2^ in male  - Skeletal Muscle index ≤38 cm^2^/m^2^ in female | **1.73 (1.52-1.96)** | **< 0.001** | | **1.24 (1.09-1.42)** | **0.001** |
| Age (every 1-year increase) | 1.07 (1.06-1.08) | < 0.001 | | 1.06 (1.05-1.07) | < 0.001 |
| Men | 0.92 (0.80-1.07) | 0.274 | | 1.06 (0.9-1.24) | 0.489 |
| Presentation with AMI | 1.57 (1.35-1.84) | < 0.001 | | 1.12 (0.96-1.32) | 0.153 |
| Hypertension | 1.43 (1.24-1.64) | < 0.001 | | 1.12 (0.97-1.3) | 0.118 |
| Dyslipidemia | 0.88 (0.77-1.01) | 0.064 | | 0.99 (0.87-1.13) | 0.891 |
| Current smoking | 0.93 (0.81-1.06) | 0.260 | | 1.15 (1.00-1.33) | 0.057 |
| Chronic kidney disease | 4.09 (3.43-4.88) | < 0.001 | | 3.02 (2.51-3.63) | < 0.001 |
| Previous stroke | 1.82 (1.55-2.13) | < 0.001 | | 1.37 (1.16-1.61) | < 0.001 |
| Previous PCI | 1.31 (1.12-1.53) | < 0.001 | | 1.32 (1.13-1.55) | < 0.001 |
| Preoperative LVEF (every 1% increase) | 0.98 (0.97-0.98) | < 0.001 | | 0.98 (0.98-0.99) | < 0.001 |
| Three-vessel disease | 1.26 (1.09-1.46) | 0.002 | | 1.11 (0.95-1.29) | 0.183 |
| Off-pump coronary artery bypass grafting | 0.46 (0.40-0.53) | < 0.001 | | 0.83 (0.71-0.97) | 0.020 |
| Lipid-lowering therapy | 0.50 (0.43-0.57) | < 0.001 | | 0.70 (0.60-0.80) | < 0.001 |

The c-statistic of the multivariable model was 0.77.

^*^ Multivariable Cox regression model was constructed using variables with P <0.1 or clinical relevance.

Abbreviations: AMI = acute myocardial infarction; CI = confidence interval; LVEF = left ventricular ejection fraction; PCI = percutaneous coronary intervention.

**Supplementary Table 3. Baseline Characteristics of the PSM-Matched Population**

|  | **Overall Population**  **(N=1,830)** | **Preserved SMI**  **(N=915)** | **Reduced SMI**  **(N=915)** | ***P* value** | **Standard Mean Difference** |
| --- | --- | --- | --- | --- | --- |
| **Age, years** | 67.6 ± 8.4 | 67.5 ± 8.1 | 67.6 ± 8.8 | 0.833 | 0.010 |
| **Male, n (%)** | 1,295 (70.8) | 652 (71.3) | 643 (70.3) | 0.681 | 0.022 |
| **Height, cm** | 162.2 ± 8.6 | 161.4 ± 8.6 | 163.0 ± 8.5 | <0.001 | 0.190 |
| **Weight, kg** | 63.1 ± 10.2 | 66.1 ± 10.3 | 60.2 ± 9.3 | <0.001 | 0.606 |
| **Body mass index, kg/m^2^** | 23.9 ± 3.0 | 25.3 ± 2.8 | 22.6 ± 2.6 | <0.001 | 1.023 |
| **Presentation with AMI** | 350 (19.1) | 170 (18.6) | 180 (19.7) | 0.593 | 0.028 |
| **Cardiovascular risk factors** |  |  |  |  |  |
| Hypertension | 1,201 (65.6) | 610 (66.7) | 591 (64.6) | 0.376 | 0.044 |
| Diabetes mellitus | 884 (48.3) | 444 (48.5) | 440 (48.1) | 0.888 | 0.009 |
| Dyslipidemia | 576 (31.5) | 292 (31.9) | 284 (31.0) | 0.725 | 0.019 |
| Current smoking | 541 (29.6) | 269 (29.4) | 272 (29.7) | 0.918 | 0.007 |
| Chronic kidney disease | 165 (9.0) | 85 (9.3) | 80 (8.7) | 0.744 | 0.019 |
| Previous stroke | 297 (16.2) | 156 (17.0) | 141 (15.4) | 0.375 | 0.044 |
| Peripheral artery disease | 132 (7.2) | 64 (7.0) | 68 (7.4) | 0.786 | 0.017 |
| Previous myocardial infarction | 141 (7.7) | 76 (8.3) | 65 (7.1) | 0.381 | 0.045 |
| Previous PCI | 314 (17.2) | 167 (18.3) | 147 (16.1) | 0.239 | 0.058 |
| Previous CABG | 19 (1.0) | 12 (1.3) | 7 (0.8) | 0.356 | 0.054 |
| **Off-pump CABG** | 1,466 (80.1) | 732 (80.0) | 734 (80.2) | 0.953 | 0.005 |

Values are expressed as mean ± standard deviation or number (%).

Abbreviations: AMI = acute myocardial infarction; CABG = coronary artery bypass grafting; HDL = high-density lipoprotein; hs-CRP = high sensitivity C-reactive protein; LDL = low-density lipoprotein; LVEF = left ventricular ejection fraction; PCI = percutaneous coronary intervention; SMI = skeletal muscle index.

**Supplementary Table 4. Comparison of Clinical Outcomes According to Reduced SMI at 14 Years (Median 8.7-Year Follow-Up)**

|  | **Preserved SMI**  **(N=1,886)** | **Reduced SMI**  **(N=924)** | **Unadjusted HR**  **(95% CI)** | ***P* value** |
| --- | --- | --- | --- | --- |
| **All-cause death** | 484 (62.8%) | 375 (41.4%) | 1.78 (1.55-2.03) | <0.001 |
| **Myocardial infarction** | 21 (97.4%) | 16 (97.9%) | 1.70 (0.89-3.26) | 0.111 |
| **Any revascularization** | 41 (97.4%) | 26 (96.7%) | 1.38 (0.84-2.25) | 0.204 |
| **Stroke** | 69 (93.6%) | 39 (94.9%) | 1.25 (0.84-1.85) | 0.271 |
| **MACCE** | 547 (58.8%) | 408 (38.8%) | 1.72 (1.51-1.96) | <0.001 |

Data expressed as event number (survival rate) at 14 years (median 8.7 years).

^*^ Adjusted for variables of age, sex, presentation with acute myocardial infarction, hypertension, dyslipidemia, current smoking, chronic kidney disease, previous stroke, previous PCI, preoperative left ventricular ejection fraction, three-vessel disease, off-pump coronary artery bypass grafting, lipid-lowering therapy.

^†^ For propensity score-matching and inverse probability treatment-weighting analysis, the logistic regression model for the presence of reduced SMI was applied to calculate propensity scores. Patients with reduced SMI were matched 1:1 with patients with preserved SMI by “nearest-neighbor matching” (a greedy match) without replacement and a caliper size predefined as 0.2. The inverse probability treatment-weighting analyses were performed based on propensity scores. Residual differences in characteristics between matched cohorts were assessed by calculating the absolute standardized mean differences. Standardized mean differences were less than 0.1 across all matched covariates (age, sex, presentation with acute myocardial infarction, hypertension, diabetes mellitus, dyslipidemia, smoking, previous stroke, previous percutaneous coronary intervention, and off-pump CABG), indicating a good balance. All confidence intervals for the inverse probability weighting analyses were assessed with bootstrapping methods with 1,000 iterations.

Abbreviations: CI = confidence interval; HR = hazard ratio; IPTW = inverse probability treatment-weighting; MACCE = major adverse cardiac and cerebrovascular event; PSM = propensity score-matching; SMI = skeletal muscle index.

**Supplementary Figure 1. Determination of the Cut-Off Value of the Skeletal Muscle Index for Predicting Death**


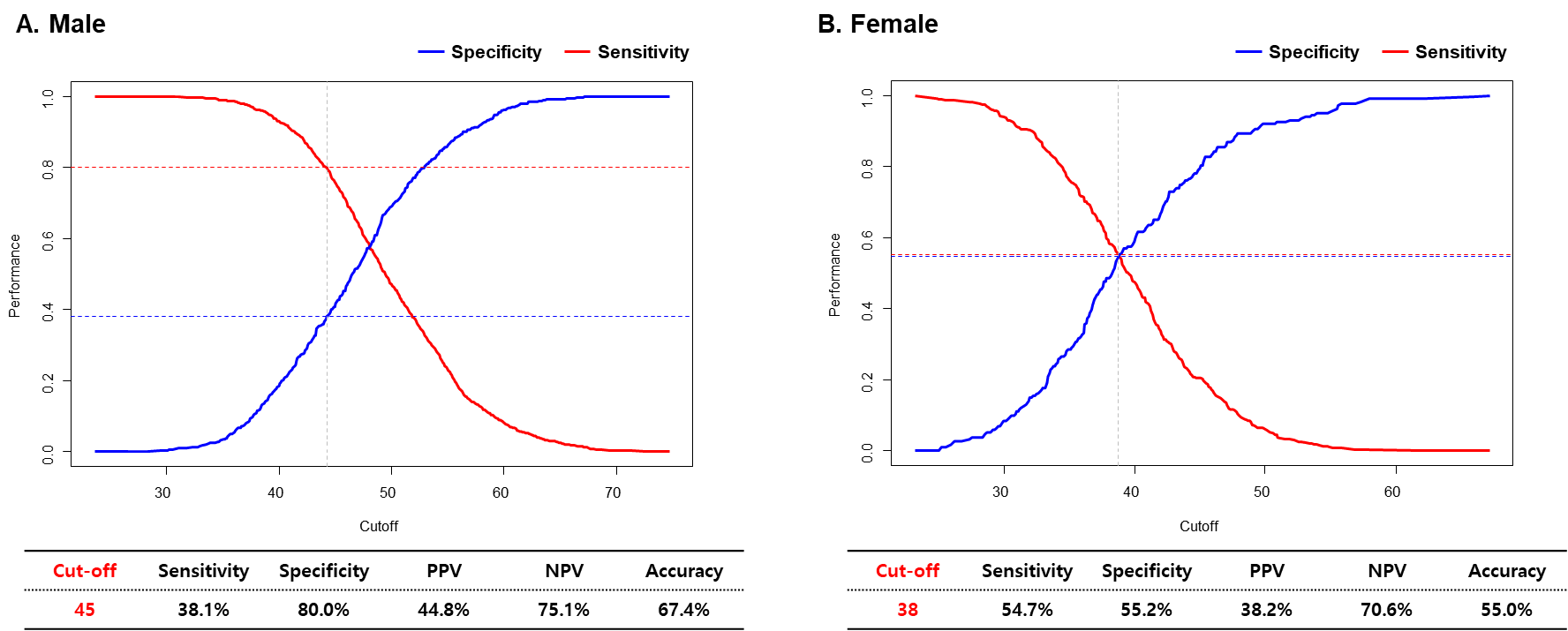


The optimal cut-off value of the skeletal muscle index for the occurrence of all-cause death was 45 cm^2^/m^2^ for male and 38 cm^2^/m^2^ for female.

Abbreviations: NPV = negative predictive value; PPV = positive predictive value.

**Supplementary Figure 2. Relationship between Skeletal Muscle Index and Fat**


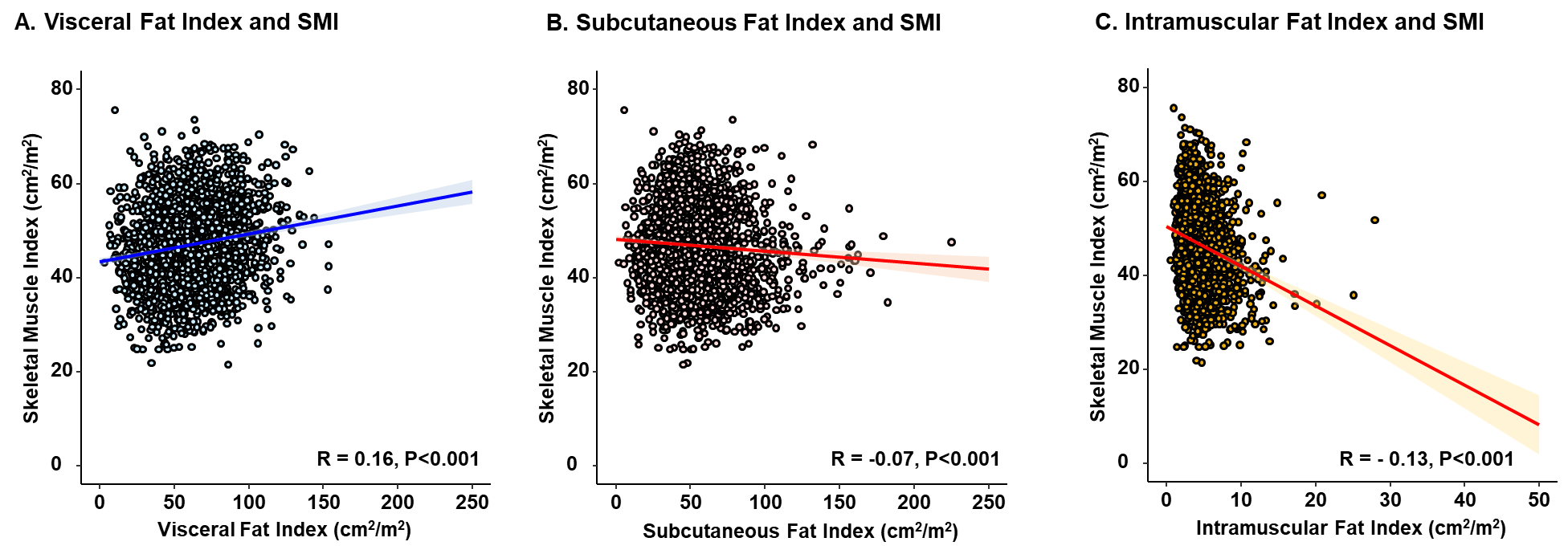


Scatter plots showing the correlation between the SMI and visceral, subcutaneous, or intramuscular fat index. Each fat area was normalized for height.

Abbreviations: SMI = skeletal muscle index.

**Supplementary Figure 3. Predicting All-Cause Death with Skeletal Muscle Index as a Continuous Variable During a Median 8.7-Year Follow-Up**


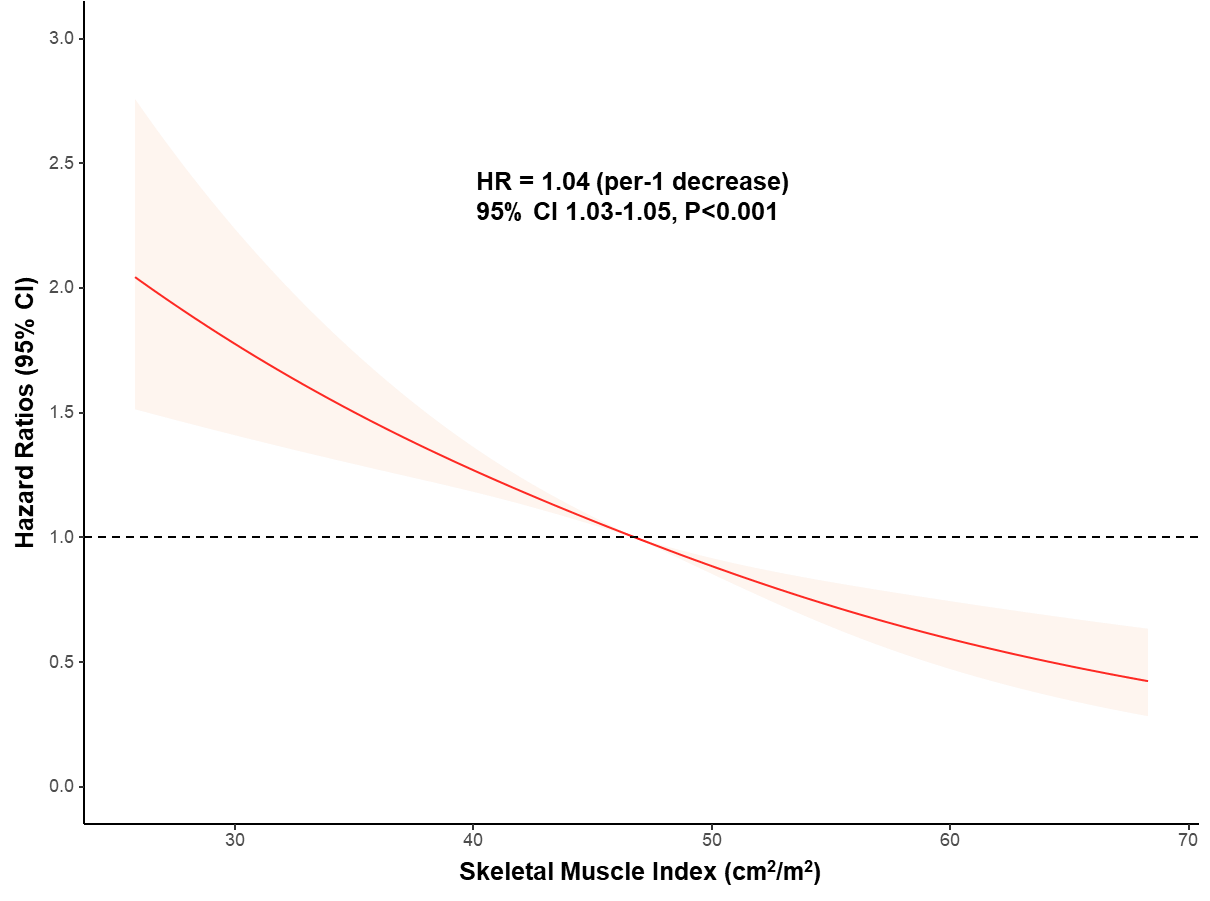


The spline curve demonstrates the association between skeletal muscle index and all-cause death risk during long-term follow-up.

Abbreviations: CI = confidence interval; HR = hazard ratio.

**References**

1. Durrleman S, Simon R. Flexible regression models with cubic splines. Stat Med. 1989;8:551-61.

2. Williamson EJ, Forbes A, White IR. Variance reduction in randomised trials by inverse probability weighting using the propensity score. Stat Med. 2014;33:721-37.

3. McCandless LC, Gustafson P, Levy A. Bayesian sensitivity analysis for unmeasured confounding in observational studies. Stat Med. 2007;26:2331-47.
